# Subthreshold Current Suppression in ReS_2_ Nanosheet-Based Field-Effect Transistors at High Temperatures

**DOI:** 10.1021/acsanm.3c03685

**Published:** 2023-11-16

**Authors:** Ofelia Durante, Kimberly Intonti, Loredana Viscardi, Sebastiano De Stefano, Enver Faella, Arun Kumar, Aniello Pelella, Francesco Romeo, Filippo Giubileo, Manal Safar G. Alghamdi, Mohammed Ali S. Alshehri, Monica F Craciun, Saverio Russo, Antonio Di Bartolomeo

**Affiliations:** † Department of Physics “E. R. Caianiello”, University of Salerno, via Giovanni Paolo II 132, Fisciano, 84084 Salerno, Italy; ‡ Department of Science and Technology, Università degli studi del Sannio, via dei mulini 59/A, Benevento 82100, Italy; § CNR-SPIN, via Giovanni Paolo II 132, Fisciano, 84084 Salerno, Italy; ∥ University of Exeter, Stocker Road 6, Exeter EX4 4QL, Devon, U.K.

**Keywords:** ReS_2_, transition-metal dichalcogenides, field-effect transistors, charge trapping, current suppression, gate current

## Abstract

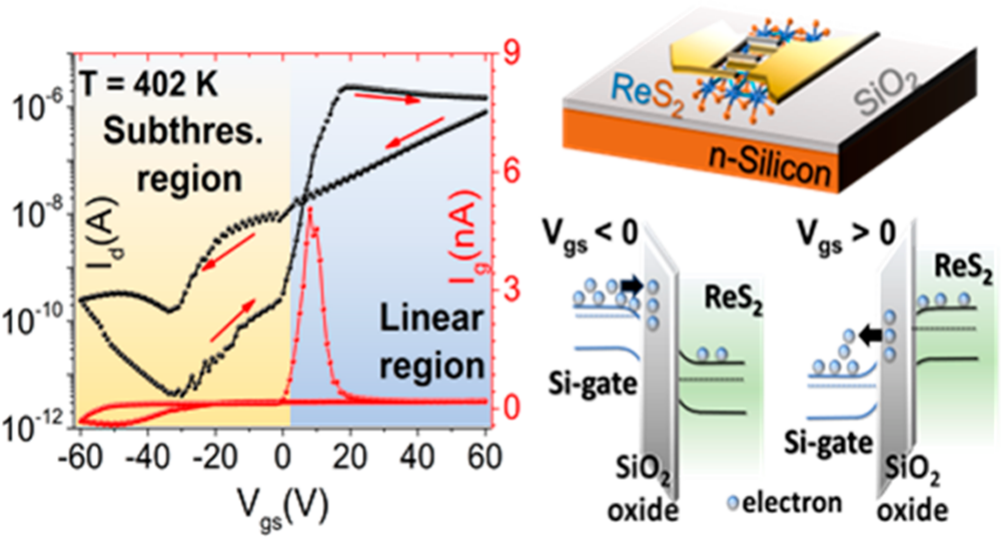

Two-dimensional rhenium disulfide (ReS_2_), a member of the transition-metal dichalcogenide family, has received significant attention due to its potential applications in field-effect transistors (FETs), photodetectors, and memories. In this work, we investigate the suppression of the subthreshold current during the forward voltage gate sweep, leading to an inversion of the hysteresis in the transfer characteristics of ReS_2_ nanosheet-based FETs from clockwise to anticlockwise. We explore the impact of temperature, sweeping gate voltage, and pressure on this behavior. Notably, the suppression in current within the subthreshold region coincides with a peak in gate current, which increases beyond a specific temperature but remains unaffected by pressure. We attribute both the suppression in drain current and the presence of peak in gate current to the charge/discharge process of gate oxide traps by thermal-assisted tunnelling. The suppression of the subthreshold current at high temperatures not only reduces power consumption but also extends the operational temperature range of ReS_2_ nanosheet-based FETs.

## Introduction

Among two-dimensional (2D) layered materials, transition-metal dichalcogenides (TMDs) have exhibited remarkable optoelectronic properties, garnering significant attention due to their tunable bandgap.^1,2^ The most widely used and exploited TMD is molybdenum disulfide (MoS_2_), characterized by a direct bandgap (∼1.85 eV) in monolayer and an indirect bandgap (∼1.2 eV) in multilayer form.^3^ These features make it suitable for sensitive photodetectors,^4^ optoelectronic memories,^5^ and performant field-effect transistors (FETs).^6−8^ Nevertheless, the exploration of TMDs is moving forward to other family members with very interesting properties.^9−11^ Distinctively, unlike materials such as graphene, which exhibit a zero bandgap, rhenium disulfide (ReS_2_) maintains a direct bandgap (∼1.5 eV) consistently in both bulk and monolayer forms. This remarkable property results from the electronic and vibrational decoupling of the stacked monolayers.^12^ Consequently, it enables tailor-made transistors for logic and optoelectronic applications, even in the face of the limited mobility of ReS_2_.

ReS_2_ adopts a triclinic symmetry characterized by S–Re–S layers held together by strong covalent bonds and weak van der Waals interactions.^13^ In contrast to TMDs in the VIB group, monolayer ReS_2_ shows a single distorted 1T structure as the stable phase. This is a result of the additional valence electron associated with the rhenium atom, which belongs to the VIIB group, leading to the formation of extra Re–Re bonds in ReS_2_.^14^ Moreover, its distinctive atomic structure, asymmetric crystal growth, and highly anisotropic monolayer configuration bestow upon ReS_2_ fundamental properties that distinguish it from other TMDs.^15^ Due to its heavy element composition, ReS_2_ demonstrates a more pronounced spin–orbit interaction than other TMDs.^15^ Although it lacks intrinsic magnetism, ReS_2_ can be induced to exhibit magnetic properties through doping or the absorption of foreign atoms, which introduce additional stray electrons. This characteristic renders it suitable for applications in the field of spintronics.^16,17^

Recently, several works have showcased the potential of ReS_2_ in high-responsivity photodetectors^18^ and field-effect transistors (FETs) with a high ON/OFF current ratio.^19^

In a study by Shim et al.,^20^ they achieved exceptional performance in a ReS_2_-based FET, demonstrating a high ON/OFF current ratio, enhanced mobility, and remarkable photoresponsivity. This was accomplished by precisely controlling the thickness of ReS_2_, reducing it from 57 to 36 nm through an O_2_ plasma treatment. Notably, low-energy O_2_ gas effectively disintegrates the weak interlayer bonds due to its low coupling energy, leading to the creation of defects (traps) on the surface of ReS_2_.

Most 2D material-based transistors exhibit a noticeable hysteresis in their transfer curve, which can be attributed to several factors, including the adsorption of oxygen or water molecules,^21,22^ the presence of oxide and interface traps,^13,14^ and the effects of gate voltage stress effects.^23^ Hysteresis can be advantageous for enabling programmable nonvolatile memories (NVMs) operations. Transistors made with monolayers or few layers of materials such as MoS_2_,^24,25^ few layers of ReS_2_,^26^ or graphene^27^ hold promise for the development of advanced NVM devices due to their distinctive physical properties. For instance, the high mobility of graphene may contribute to faster operation speeds.^27,28^ This holds true for black phosphorus (BP) as well, which enables p-type FETs with mobility of a few hundred cm^2^ V^–1^ s^–1^.^29,30^ Furthermore, temperature plays a crucial role in NVMs. Operations that require greater power per data storage can elevate the operating temperature of data centers, leading to reliability concerns.^31^ Recently, Kumar et al. reported a high-temperature stable memory using a few-layered black phosphorus with two current states (erase and program states) becoming more separated at higher temperatures.^32^ Consequently, the potential to employ a wide operational temperature range in 2D material-based devices is a groundbreaking development in the field of memory. Goyal et al.^26^ demonstrated a thermally assisted memory using back-gated FETs made of few-layer ReS_2_ and MoS_2_. They observed a step-like increase in drain current with rising temperature in the transfer curve for both materials. This spontaneous feature, which occurs at a slightly lower temperature for ReS_2_ (373 K) than for MoS_2_ (400 K), can facilitate the reliable and controlled storage of binary data by exploiting different current states. Additionally, temperature significantly impacts the current in the subthreshold region of an FET. As the temperature increases, the subthreshold leakage current tends to rise, impacting power consumption and overall device efficiency.^33^ Therefore, any reduction in the subthreshold current is highly advantageous.

In this work, we present a ReS_2_ nanosheet-based FET device that exhibits a remarkable suppression in the drain current within the subthreshold region during the forward gate voltage sweep. Additionally, it exhibits a crossover in current behavior between forward and backward gate voltage sweeps, with these effects becoming particularly pronounced at temperatures exceeding 360 K. Interestingly, the gate current demonstrates a peak at the same gate voltages where the drain current returns to its normal values, after being suppressed. The intensity of this peak increases with temperature and is influenced by the range of sweeping gate voltages. Furthermore, under low-pressure conditions, where the desorption of oxygen and water leads to an increase in drain current, we observe the same correlation between the end of drain current suppression and the emergence of the peak in the gate current above 360 K. The suppression of the subthreshold current in ReS_2_ nanosheet-based FETs at high temperatures can be a great operational advantage and is attributed to thermally assisted charging/discharging of the SiO_2_ gate oxide layer.

## Experimental Section

### Device Fabrication

**Figure 1:**
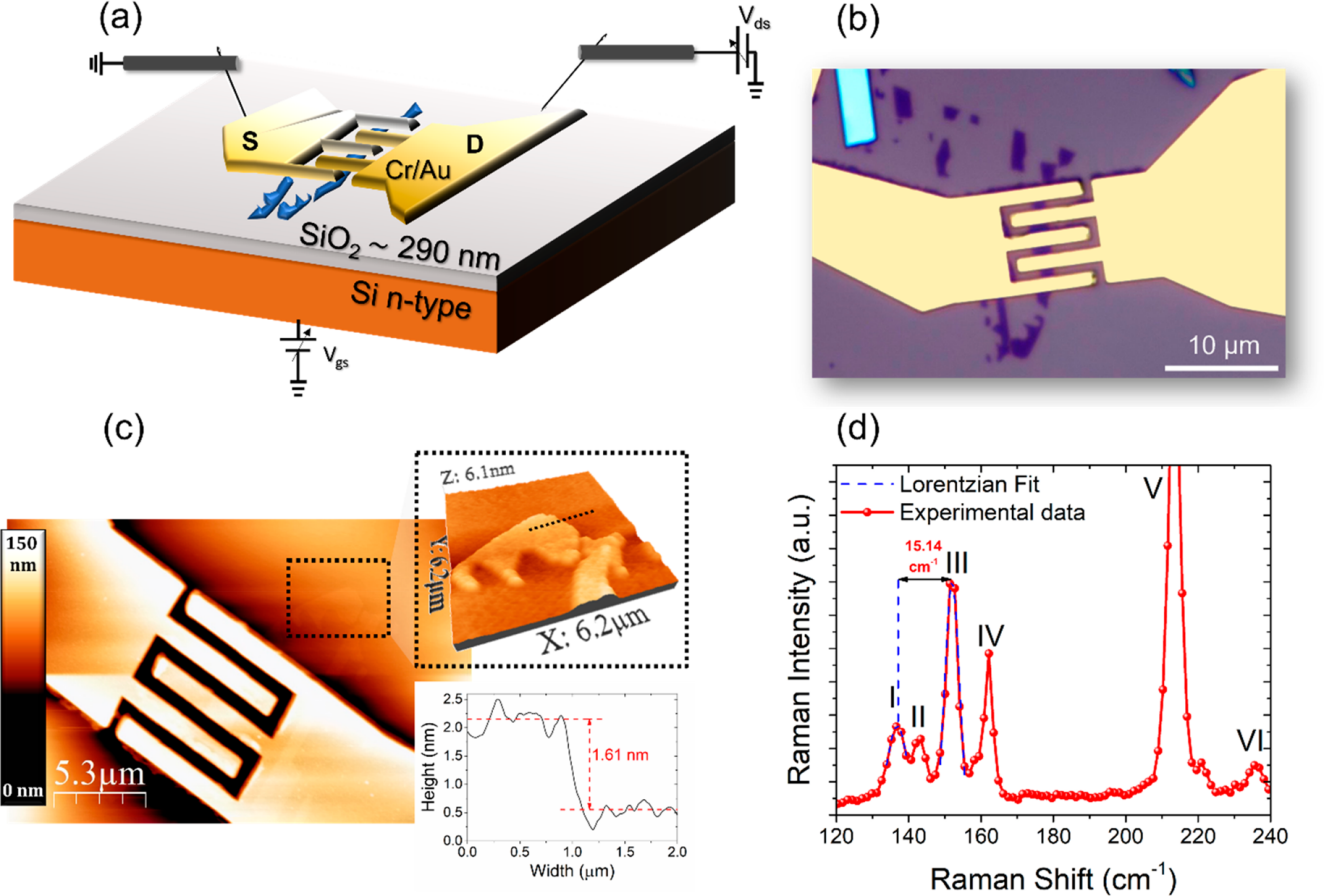
(a) Schematic diagram of the back-gate FET interdigitated device with a ReS_2_ flake as the channel, metal leads as the source (S) and drain (D), and the Si substrate as the common back-gate. (b) Processed optical image of ReS_2_ flakes contacted with Cr/Au leads. (c) AFM image of the ReS_2_ nanosheet-based FET. The inset shows a 3D-image of a selected area (top) and the height profile of the ReS_2_ flake (bottom). (d) Raman spectrum of the ReS_2_ flakes (red curve) and Lorentzian fit (blue dashed curve) of Raman modes I and III.

ReS_2_ flakes were exfoliated from a bulk ReS_2_ single crystal and transferred onto a highly doped n-type Si substrate with a resistivity ranging from 0.001 to 0.005 Ω cm. The Si substrate is covered with a 290 nm layer of thermal SiO_2_. These flakes are contacted by metal interdigitated electrodes of Cr (5 nm) and Au (110 nm), used as adhesion and cover layers, respectively. The metallic materials are deposited by thermal evaporation to minimize the contact resistance.^34^Figure 1a shows a schematic representation of the back-gate FET formed by the ReS_2_ flake as the channel, while the Cr/Au interdigitated leads are the source (S) and the drain (D). The highly doped Si substrate is used for the common back-gate. An optical image in Figure 1b offers a top view of the device, revealing the rectangular shape of the ReS_2_ flake and the interdigitated design of the metal contacts. The design comprises four parallel channels, each with a width (*W*) of 1.25 μm and a length (*L*) of 1 μm. Figure 1c shows the topographic Atomic Force Microscopy (AFM) image of the device, revealing a thickness of ∼1.61 nm for the flake typical of bilayer ReS_2_ (see Supporting Information for more details). Owing to the significant thickness of the metallic leads, we measured the height profile on the portion of flake outside the device (see upper inset), obtaining a thickness of ∼1.61 nm, corresponding to two layers of ReS_2_.^35^

Figure 1d shows a Raman spectrum measured on the bilayer (see Supporting Information for more details). Several high-energy Raman modes of ReS_2_, labeled as I–II (A_g_-like vibrational modes) and III–VI (E_g_-like vibrational modes), are clearly observed. These modes can be employed to determine the thickness of the flake. Specifically, the difference between the positions of modes III and I provides a clear indication of the thickness of ReS_2_. Comparing this difference, which is measured as 15.14 cm^–1^ and obtained through Lorentzian fits (indicated by the blue dashed curve), with values reported in the literature^14,36,37^ confirms that the flake consists of a bilayer structure.

## Results and Discussion

**Figure 2:**
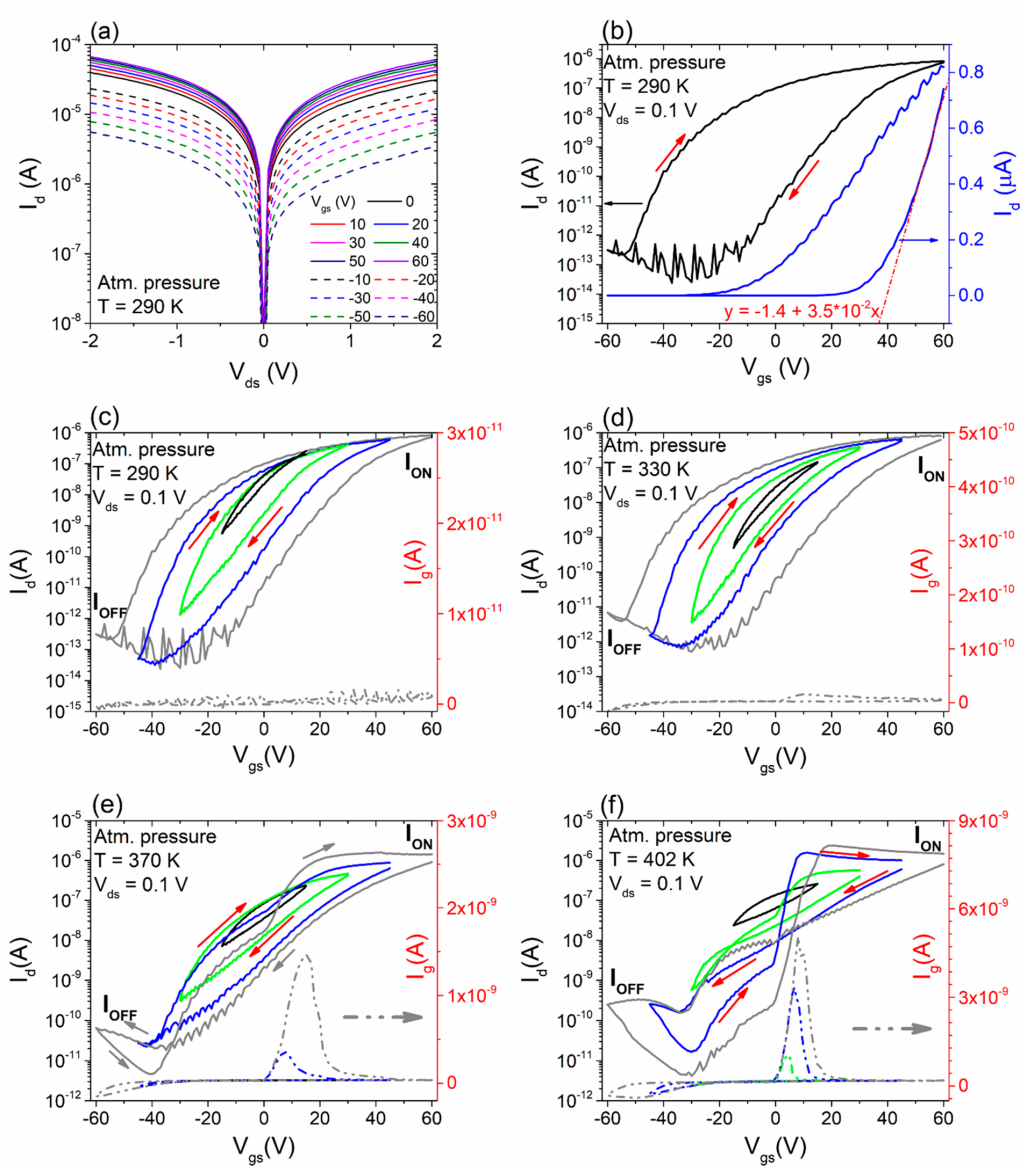
Room temperature and atmospheric pressure: (a) Output characteristic at different values of *V*_gs_; (b) Transfer characteristics at *V*_ds_ = 0.1 V for a forward and a reverse *V*_gs_ sweep, on logarithmic (black curve) and linear (blue curve) scale (the dashed red line is the linear fit). Transfer curves at atmospheric pressure for different temperatures: (c) 290, (d) 330, (e) 370, and (f) 402 K with gate current control (colored dashed curves).

The standard characterization of the ReS_2_ nanosheet-based transistor was initially performed under atmospheric pressure. Figure 2a shows the output curves, specifically drain current vs drain voltage, *I*_d_–*V*_ds_, for different gate voltages *V*_gs_ ranging from −60 to 60 V. In these output curves, *I*_d_ varies linearly with the bias *V*_ds_, exhibiting ohmic contact behavior for positive (solid-colored curves) and negative (dashed-colored curves) *V*_gs_ values. The presence of an ohmic behavior indicates a negligible Schottky barrier at the ReS_2_–Cr/Au interface, as expected. This outcome aligns with expectations because Cr possesses a work function of 4.5 eV, which is lower than that of ReS_2_ (∼4.8 eV) and close to the conduction band minimum of ReS_2_ (∼4.3 eV).^38,39^ The behavior observed in Figure 2a is typical of a n-type device, where a positive (negative) back-gate voltage enhances (reduces) the charge carrier density resulting in an increase (decrease) of the drain current. Several works on both monolayer and few-layer ReS_2_ FET devices corroborate our findings, reporting a linear response but with on-current lower than that shown in Figure 2a.^39,40^ The transfer characteristics (*I*_d_–*V*_gs_ for fixed *V*_ds_) shown in Figure 2b confirm the n-type behavior of the ReS_2_-based FET characterized by a high ON/OFF ratio (∼10^7^) and a low off-state current (10^–13^ A) in the range of *V*_gs_ = ±60 V with a voltage bias of 0.1 V.

The blue curve in Figure 2b represents the transfer characteristic on a linear scale. The field effect electron mobility, μ, can be estimated by analyzing the linear regime of the transfer curve using the expression , where *C*_ox_ = 1.15 × 10^–8^ F cm^–2^ is the gate dielectric capacitance per unit area, *L* and *W* are the channel length and width, respectively, and the coefficient 4 accounts for the number of channels in the interdigitated device. As reported in ref (40), the electron mobility in ReS_2_ increases with a reduction in the number of layers, which may explain the lower mobility compared to that reported elsewhere for monolayer ReS_2_.^41,42^ We note also that the obtained mobility is comparable to that reported for other TMDs used in similar devices.^6,43−45^

The effect of the temperature and the sweeping gate voltage range is illustrated in Figure 2c–f. For each temperature, we present the transfer characteristics of the device at constant drain bias *V*_ds_ = 0.1 V and for various sweeping gate voltage ranges (in different colors), namely, (−15 V, 15 V), (−30 V, 30 V), (−45 V, 45 V), and (−60 V, 60 V). To avoid gate oxide damage, we avoided applying gate voltages higher than 60 V.

In Figure 2c, the transfer characteristic at room temperature confirms the n-type behavior of the device. It exhibits an enhancement of the on-current *I*_ON_ and simultaneously a decrease of the off-current *I*_OFF_ as well as an increase in the hysteresis width of the transfer curve with increasing sweeping gate voltages.^23^ The large current is a consequence of the wide channel and the low resistance of the ohmic contacts. A clockwise (CW) hysteresis is observed, as highlighted by the red arrows in Figure 2c.

The widening hysteresis loop, observed with an increasing *V*_gs_ sweeping range, is attributed mainly to trapping and detrapping from localized intragap states resulting from impurities or S vacancies in the ReS_2_ flake.^46,47^ The presence of oxygen and water adsorbates also contributes to this hysteresis.^48,49^ Noteworthy, in all (c–f) panels of Figure 2, we report the gate current (*I*_g_) acquired as a function of the gate voltage (see colored dashed curves), simultaneously with the drain current. We observe that at 290 K, the gate current remains constant around 0.1 pA (see dashed gray curve in panel c). Figure 2d shows the transfer curves for different *V*_gs_ sweeping ranges at the temperature of 330 K. The n-type characteristics and a slight increase in the drain current, *I*_ON_, are observed; however, the shapes and the hysteresis width behavior of the transfer curves are like those observed at 290 K. Conversely, a change in the aspect of the hysteretic loop is observed at higher temperatures. Figure 2e shows the transfer curves at 370 K. We observe an enhancement of *I*_ON_ and the gate current by increasing the temperature and the gate sweeping range. Notably, the curves with larger *V*_gs_ sweeping ranges (blue and gray curves) exhibit an anomalous behavior. Specifically, at high temperatures, a suppression of the drain current occurs during the forward *V*_gs_ sweep from −45 to about 0 V (blue curve) or from −60 to 10 V (gray curve). In both cases, when the suppression in the drain current occurs, a peak in the gate current (colored dashed curves) at higher *V*_gs_ is observed. We point out that this feature holds significant importance from a device standpoint as it enables low static power consumption at high temperatures and voltage biases. The increase in subthreshold current with temperature and bias represents a critical issue in modern field-effect devices. Consequently, this finding can open new perspectives in the use of 2D devices in electronic applications.

To investigate this feature further, we further increased the temperature up to 402 K (see panel f). In this scenario, for a sweeping gate voltage (−30, 30 V), the current suppression is already observed (see green curve), and its end corresponds to a peak in the gate current. Similarly, a suppression in the drain current and a peak in the gate current are observed for an increased sweeping gate voltage. At 402 K, we also observe a crossover of the *I*_d_ curves in the forward and reverse sweeping directions (highlighted by red arrows in panel f). In particular, the crossover of the drain current moves from positive to negative gate voltages for different sweeping gate voltage ranges. For instance, the crossing occurs at *V*_gs_ = −16 V for −30 V ≤ *V*_gs_ ≤ 30 V, at *V*_gs_ = 1 V for −45 V ≤ *V*_gs_ ≤ 45 V, etc. Consequently, a transition from CW hysteresis at 290–370 K to partially anticlockwise (ACW) hysteresis at 402 K can be observed in the transfer curves of the ReS_2_ nanosheet-based FETs.

Furthermore, we performed electrical measurements at 2.3 mbar (see Supporting Information) and observed a higher drain current and greater mobility compared with measurements at atmospheric pressure. However, we noted that the same decrease in the drain current correlated to a peak of the gate current, occurring within the temperature range 350–360 K. Therefore, we concluded that the drain current suppression in ReS_2_ transistors is not influenced by changes in pressure.

The phenomenon of current suppression can be explained by charge trapping occurring in the gate oxide. It is well-established that SiO_2_ possesses defects, interfacial states, or impurities that promote charge trapping. The trap dynamics is significantly temperature-dependent. Higher temperatures provide additional energy to charges, facilitating both the filling of empty trap sites and the escape from filled trap sites. Conversely, at lower temperatures, charge trapping is slower, and trapped charges are retained for longer periods. In fact at these temperatures, the carriers in the gate lack the necessary energy to access the deep traps within the oxide.^25,26^

**Figure 3:**
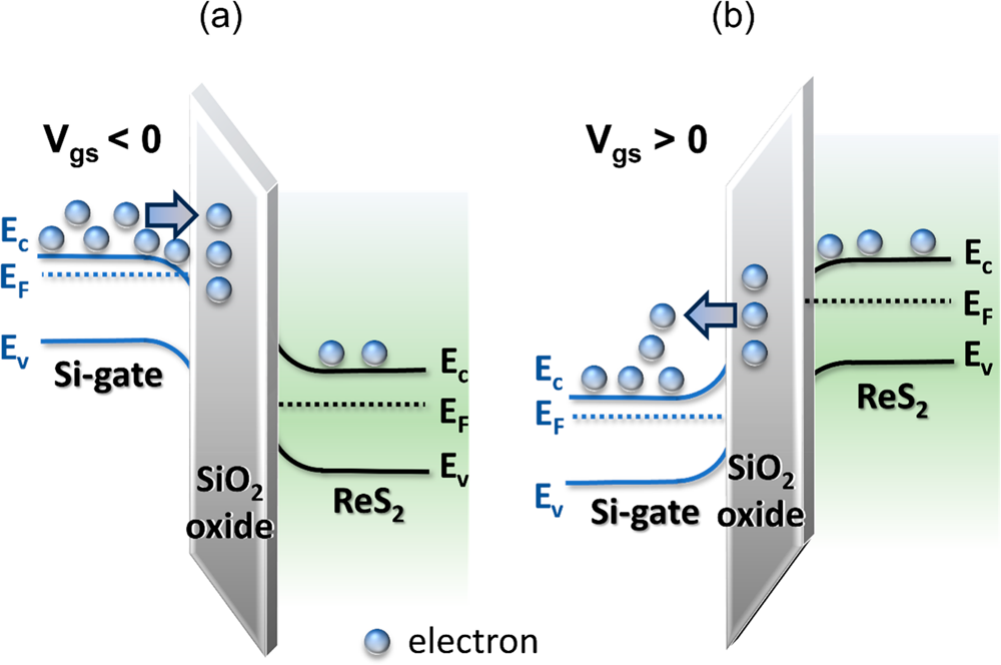
Band diagrams of the ReS_2_ nanosheet-based FETs at high temperature for (a) *V*_gs_ < 0 V and (b) *V*_gs_ > 0 V with charge trapping/detrapping.

Consequently, we can attribute the drain current suppression at high temperature, shown in Figure 2f, to the trapping and detrapping of electrons in the gate oxide SiO_2_, as previously suggested for similar devices.^25,26^ When a negative gate voltage (*V*_gs_ < 0 V) is applied, the energy bands of the Si/SiO_2_/ReS_2_ structure undergo a bending effect, as depicted in the diagram presented in Figure 3a. This bending facilitates the transfer of electrons from the Si-gate to the SiO_2_-oxide layer during the negative sweep.

**Figure 4:**
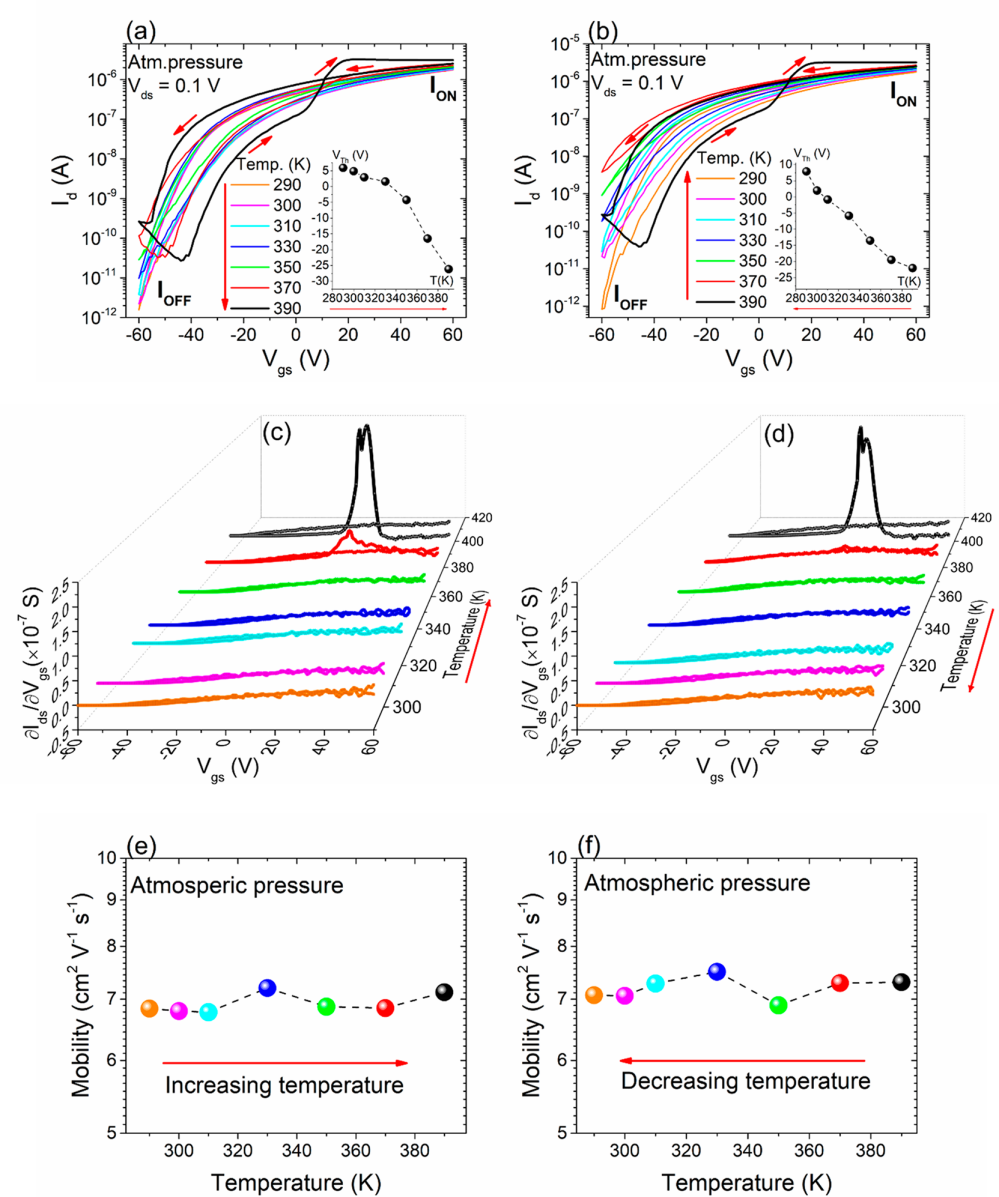
Atmospheric pressure: transfer characteristics at *V*_ds_ = 0.1 V by (a) increasing and (b) decreasing the temperature. (inset) *V*_th_ vs temperature. Transconductance curves vs *V*_gs_ by (c) increasing and (d) decreasing the temperature with a sweeping gate voltage from −60 to +60 V. Mobility extracted from the transfer curves at different temperature directions, i.e., (e) increasing and (f) decreasing.

The electrons trapped in the oxide layer generate a repulsive force on the free carriers, resulting in current suppression within the ReS_2_ channel. However, when the gate-source voltage becomes positive (*V*_gs_ > 0 V), the energy bands change, as illustrated in Figure 3b. The new band bending facilitates the emission of the trapped electrons that, activated at high temperatures, move from the oxide back to the gate. This motion of electrons leads to a substantial increase in the gate field on the ReS_2_ channel, which is not screened anymore, thereby causing a step-like enhancement in the drain current. The charge transfer from SiO_2_ to Si originates from the observed gate current peak. The charge/discharge process within the gate oxide is a thermally assisted tunneling. To gain further insights, we focus on the temperature at which the suppression and the CW–ACW transition occur as well as the mobility concerning the direction of temperature change. Figure 4 shows the transfer curves at *V*_ds_ = 0.1 V by (a) increasing and (b) decreasing the temperature from 290 to 390 K. By increasing temperature (see panel a), no change in both the shape and the slope of the transfer characteristic is observed up to 370 K. It is at this point that current suppression and the transition from CW to ACW behavior become evident. Similarly, when we decrease the temperature, although the step is most pronounced at 390 K, the overall behavior remains consistent. This similarity in the two directions of temperature change is clearly noticeable when examining the transconductance, , as depicted in Figure 4c,d. The end of the suppressed drain current versus *V*_gs_ corresponds to a distinct peak in transconductance.

Moreover, from transfer curves corresponding to the forward *V*_gs_ sweep, we can extract the threshold voltage (*V*_th_) (see the insets of Figure 4a,b), which is the voltage required to switch the transistor from the off to on state. The decreasing values of *V*_th_ as the temperature rises suggest that the electron trapping becomes less effective at higher temperatures as trapped electrons induce a right shift of the transfer curve. The negative value of *V*_th_ indicates the natural n-doping of the ReS_2_,^18,50^ and a negligible (or very low) Schottky barrier at the Cr/Au-ReS_2_ interface.

Finally, Figure 4e,f shows the field-effect electron mobility, μ, in the case of increasing (e) and decreasing (f) temperature. Given the nonstandard behavior of the transfer curves, we chose to evaluate the mobility using the forward branch, as per the previous formula. In both cases, we observed a constant behavior over the temperature range between 290 and 390 K. The obtained mobility aligns with results from other studies involving similar devices based on different 2D materials.^20,41,43,51^

## Conclusions

In this work, we investigated ReS_2_ nanosheet-based FETs, in back-gate configuration, utilizing Cr/Au interdigitated leads. A noteworthy observation is the occurrence of a suppressed drain current within the subthreshold region at high temperatures during the forward gate voltage sweep. This suppression coincides with a peak in the gate current and manifests within the temperature range 350–370 K, remaining unaffected by changes in pressure. We attributed these phenomena to charge trapping in the gate oxide through thermally assisted tunneling.

We point out that the increase in the subthreshold current with rising temperature and drain bias represents a serious issue in modern field-effect devices. Therefore, the observed suppression of the subthreshold current constitutes a valuable outcome, enabling reduced static power consumption and efficient device operation under high-temperature and voltage bias conditions.

## Supporting Information Available

Experimental methods, highlighting the instruments used, and insights into the effect of pressure (2.3 mbar) on the suppression of subthreshold current and gate current in ReS_2_ nanosheet-based FETs (PDF)
