# Supplementary material for: Subthreshold Current Suppression in ReS2 Nanosheet-Based Field-Effect Transistors at High Temperatures
Source: ACS Appl Nano Mater. 2023 Nov 16;6(23):21663–70. doi: 10.1021/acsanm.3c03685 (PMC10714311; doi:10.1021/acsanm.3c03685)
Supplement: Supplementary file 1 — an3c03685_si_001.pdf [file an3c03685_si_001.pdf]

Supporting information

Subthreshold Current Suppression in ReS_2 Nanosheet-Based Field-Effect Transistors at High Temperatures

Ofelia Durante[1], Kimberly Intonti[1], Loredana Viscardi[1], Sebastiano De Stefano[1], Enver Faella[1], Arun Kumar[1], Aniello Pelella[2], Francesco Romeo[1], Filippo Giubileo[3], Manal Safar G. Alghamdi[4], Mohammed Ali S. Alshehri[4], Monika Craciun[4], Saverio Russo[4], and Antonio Di Bartolomeo[1,]*

1. Department of Physics "E. R. Caianiello", University of Salerno, via Giovanni Paolo II 132, Fisciano 84084, Salerno, Italy.
2. Department of Science and Technology, Università degli studi del Sannio, via dei mulini 59/A, Benevento 82100, Italy
3. CNR-SPIN, via Giovanni Paolo II 132, Fisciano 84084, Salerno, Italy
4. University of Exeter, Stocker Road 6, Exeter EX4 4QL, Devon, UK

**Table of contents**	**Pages**
Experimental Section/Methods	S2
Conductivity at 2.3 mbar and different temperatures	S2
Figure S1	S4
References	S5

*Corresponding author, Email address: adibartolomeo@unisa.it

Experimental Section/Methods

ReS_2 flakes were exfoliated from a bulk ReS_2 single crystal and transferred over a highly doped n-type Si substrate (resistivity 0.001-0.005 Ω cm) covered by 290 nm thermal SiO_2. The metal leads are fabricated by using standard photolithography and lift-off processes of evaporated Cr/Au (5 nm/110 nm) as shown in Figure 1a. The AFM image of ReS_2 flakes is acquired by a Nanosurf A.G AFM (see Figure 1c). The Raman spectra are performed with a Raman Spectrometer (RENISHAW InVia). All measurements are performed with an excitation wavelength of 532 nm, with an output power of 10% of incident laser, and grid sizes of 1800 gmm^{-1} (see Figure 1d in the main text).

The electrical measurements on ReS_2 nanosheet-based FETs are carried out in two-probe configuration, connected to a Keithley 4200 semiconductor characterization system (Tektronix Inc.) in a Janis ST-500 Probe Station (Lake Shore Cryotronics) provided with nanoprobes connected to the source/drain leads. The vacuum at 2.3 mbar is achieved through a rotary pump and controlled by a pressure gauge.

Conductivity at 2.3 mbar and different temperatures

To investigate the effects of pressure on suppression of the subthreshold current in ReS_2 nanosheet-based FETs, we performed electrical measurements at a pressure of 2.3 mbar. Figure S1a shows the output characteristics at 2.3 mbar and room temperature, for gate voltages ranging from -60 to 60 V, with a step of 10 V. Also in this case, the I_d *vs* V_{ds} curves are linear, showing the formation of good ohmic contacts [1]. The slight increase of drain currents in vacuum in the output curves, see panel a, is caused by desorption of adsorbates such as O_2 and H_2O which act as p-dopants [2].

Furthermore, as already demonstrated for other 2D materials [3], the desorption of adsorbates at low pressure causes a left shift in the transfer curve, which is shown in panel b), and a

reduction in the threshold voltage (V_{th} ~ - 53 V), related to the increase of n-type doping of the materials. A slight increase in the mobility, compared to the ambient pressure, is also observed. In fact, we calculated the mobility at ambient pressure, finding the value of $\mu = 8\ cm^2\ V^{-1}s^{-1}$ that is consistent with the fact that a lower pressure reduces adsorbates on the surface of the ReS_2 channel that act as scattering centres [4].

We investigated the gate current behavior during the acquisition of the drain current. The gate current at 2.3 mbar, as we would expect, is also temperature dependent. We observed a similar peak as at atmospheric pressure, that increases when the temperature rises. In fact, the peak goes from ~10^{-11}A at 290 K to ~10^{-9}A at 402 K, just like at ambient pressure (Figures S1c-d). The gate current is not influenced by the pressure because it is related to the charge/discharge of the gate oxide (see model in the main text).

Finally, also at 2.3 mbar, we are interested in investigating the temperature above which the subthreshold current suppression and the transition CW – ACW occurs. Figure S1e shows the transfer curves at V_{ds} = 0.1 V by increasing the temperature from 290 to 390 K. Like at atmospheric pressure, the shape and slope of the transfer curves do not change up to 360 K, where a suppression and a CW - ACW transition are observed (see red arrows). This is also confirmed by the peak in the transconductance vs V_{gs} shown in the inset of Figure S1f.

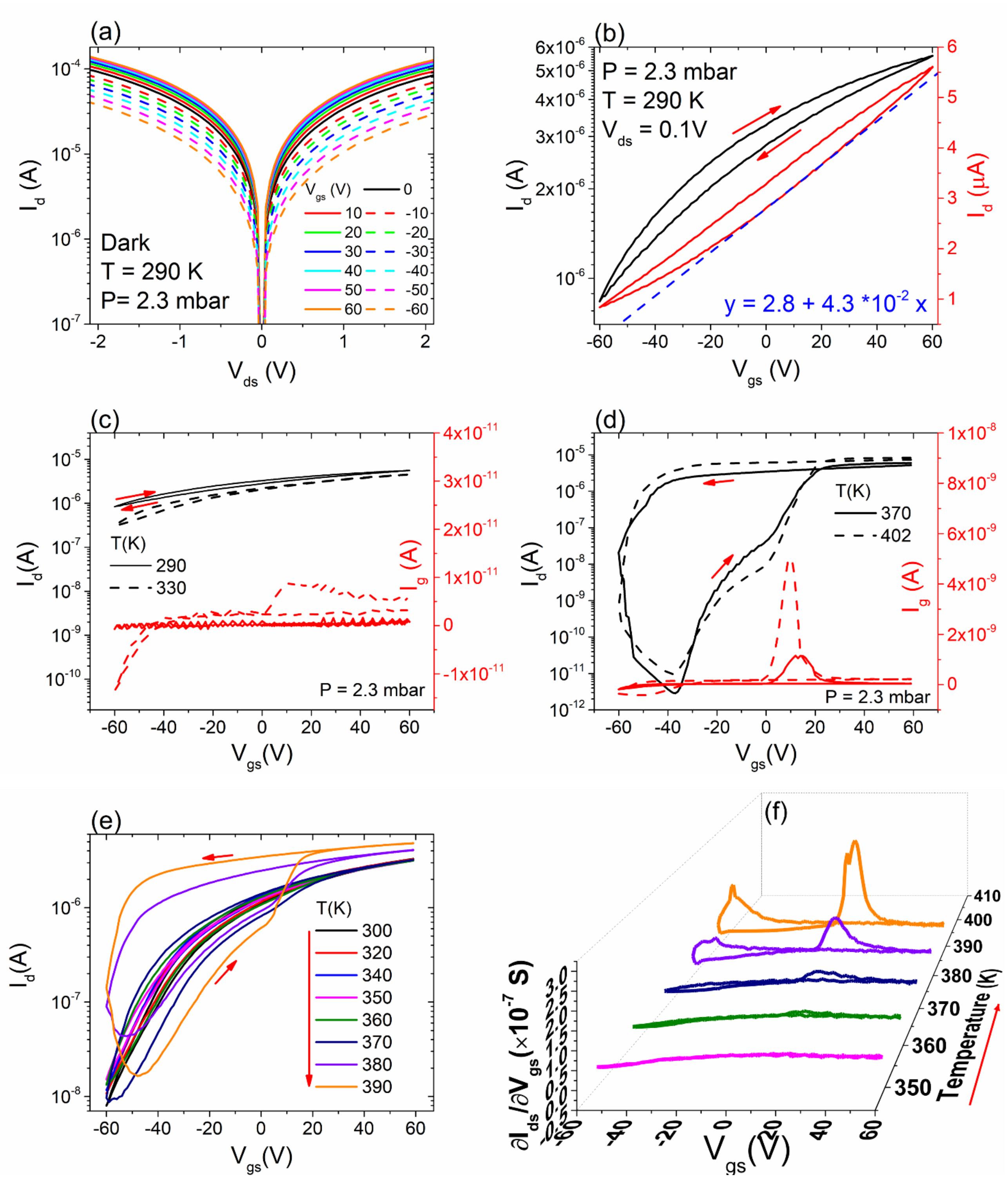

Figure S1. Pressure of 2.3 mbar: (a) output characteristic at different value of V_{gs}. (b) Transfer curve at V_{ds} = 0.1 V in log (black) and linear (red) scale at 290 K. Transfer curves at (c) 290 K (solid) and 330 K (dashed), (d) 370 K (solid) and 402 K (dashed) with gate current control (solid and dashed red curves). (e) Transfer curves at different temperatures (300-390 K). (f) Transconductance vs V_{gs} at different temperatures (350-390 K).

References

(1) Jariwala, B.; Voiry, D.; Jindal, A.; Chalke, B. A.; Bapat, R.; Thamizhavel, A.; Chhowalla, M.; Deshmukh, M.; Bhattacharya, A. Synthesis and Characterization of ReS_2 and $ReSe_2$ Layered Chalcogenide Single Crystals. *Chem. Mater.* **2016**, *28* (10), 3352–3359. https://doi.org/10.1021/acs.chemmater.6b00364.

(2) Xu, K.; Deng, H.-X.; Wang, Z.; Huang, Y.; Wang, F.; Li, S.-S.; Luo, J.-W.; He, J. Sulfur Vacancy Activated Field Effect Transistors Based on ReS_2 Nanosheets. *Nanoscale* **2015**, *7* (38), 15757–15762. https://doi.org/10.1039/C5NR04625D.

(3) Faella, E.; Intonti, K.; Viscardi, L.; Giubileo, F.; Kumar, A.; Lam, H. T.; Anastasiou, K.; Craciun, M. F.; Russo, S.; Di Bartolomeo, A. Electric Transport in Few-Layer $ReSe_2$ Transistors Modulated by Air Pressure and Light. *Nanomaterials* **2022**, *12* (11), 1886. https://doi.org/10.3390/nano12111886.

(4) Ahn, J.-H.; Parkin, W. M.; Naylor, C. H.; Johnson, A. T. C.; Drndić, M. Ambient Effects on Electrical Characteristics of CVD-Grown Monolayer MoS_2 Field-Effect Transistors. *Sci Rep* **2017**, *7* (1), 4075. https://doi.org/10.1038/s41598-017-04350-z.
